# Supplementary material for: Molecular strategies for antibody binding and escape of SARS-CoV-2 and its mutations
Source: Sci Rep. 2021 Nov 5;11:21735. doi: 10.1038/s41598-021-01081-0 (PMC8571385; doi:10.1038/s41598-021-01081-0)
Supplement: Supplementary file 1 — Supplementary Information. [file 41598_2021_1081_MOESM1_ESM.pdf]

Molecular strategies for antibody binding and escape of SARS-CoV-2 and its mutations
Supplementary Information

Mohamed Hendy, Samuel Kaufman and Mauricio Ponga

September 23, 2021

1 PMF as a function of the reaction coordinate for ACE2 and hNAbs

Fig. S1 shows the potential of mean force (PMF) for receptor binding domain (RBD) and the ACE2 receptor and several human neutralizing antibodies (hNAbs) as a function of the separation distance, λ. The shaded area represents the error estimation in the measure obtained from 200 estimations of the PMF.

The evolution of the PMF for the RBD and ACE2 receptor is found in **Fig. S1**(a). We observed how δ, MT2 and MT3 have higher binding energy compared to the WT. For MT1, we found the binding energy to be below the WT.

The PMF evolution as a function of the reaction coordinate for B38 is illustrated in **Fig. S1**(b), including the RBD with the WT, MT1, and MT3. We observed a well-defined equilibrium configuration for B38 −for the original and mutated RBD− with a global minimum at $\lambda = 0.1$ nm. The energy increased almost quadratically up to $\lambda = 0.2$ nm. After that, the PMF increased with irregular fluctuations due to the loss of contacts between the two molecules. For the mutated version, the PMF shows another local minimum around $\lambda = 0.25$ nm due to intermittent contacts on and off. These two minimum configurations are separated by an energy barrier of ~ 25 kJ·mol^{-1}. The behavior for the MT1 and MT3 versions is then similar, although the actual values differ. The PMF plateaus at around $\lambda = 1.2$ nm except for MT1.

The PMF evolution for C102 is shown in **Fig. S1(c)**, where a non-convex energy landscape with two minima (one global at $\lambda = 0.1$ nm and another local at $\lambda = 0.2$ nm) for both the original RBD and the mutated one can be observed. This behavior indicates multiple equilibrium configurations of the antibody C102 separated by a small barrier of ~ 10 kJ·mol^{-1}. After that, the PMF increased with the reaction coordinate until the proteins were separated entirely. This separation was manifested in the PMF plot with a plateau at $\lambda = 2.0$ nm and $\lambda = 1.75$ nm for C102 with the RBD in its original and mutated versions (MT1 and MT3).

Next, let us analyze the PMF for Class II P2B-2F6 hNAb, as shown in **Fig. S1(d)**. We found well-defined minimum configurations near $\lambda = 0.1$ nm for both antibodies, denoting a robust binding configuration. The PMF increased sharply for P2B-2F6 as λ increased until it reached $\lambda \sim 0.7$ nm. After this point, the PMF plateaus. The behavior for MT2 is similar, although the PMF shows a first plateau region, and then it declines. We have taken the binding energy as the average value of the first plateau.

For C144 hNAb, another one belonging to Class II, the PMF evolution also increased sharply until $\lambda \sim 0.5$ nm (see **Fig. S1(e)**). Then, the PMF showed periods with more or less constant values followed by incremental PMF regions. This behavior indicates sudden detachment of molecules as λ increased. Remarkably, when $\lambda \sim 2$ nm, the binding energy plateaued. This observation suggests that Class II has a very well-defined binding energy level, above the levels found for the ACE2 receptor and Class I hNAbs. For MT2 and MT3, we observe that the PMF had a significant lower value. We see that the most significantly decline in the PMF is given for MT2, which involves E484K and N501Y. The decrease in the PMF for MT3 shows the effect of K417N. Nevertheless, these values are well below the binding energy found for C144 and WT RBD.

We finally focus on the PMF for C135. We observed almost a monotonically increasing behavior. The actual physical detachment occurred at $\lambda \sim 2$ nm, where the PMF seemed to have an almost constant value. We have taken the binding energy as the average over that region.

The histograms for PMF sampling between the ACE2 receptor and RBD (WT, MT1, MT2, and MT3) are shown in **Fig. S2**. The histogram for δ are omitted here but similar or better space resolution was obtained.

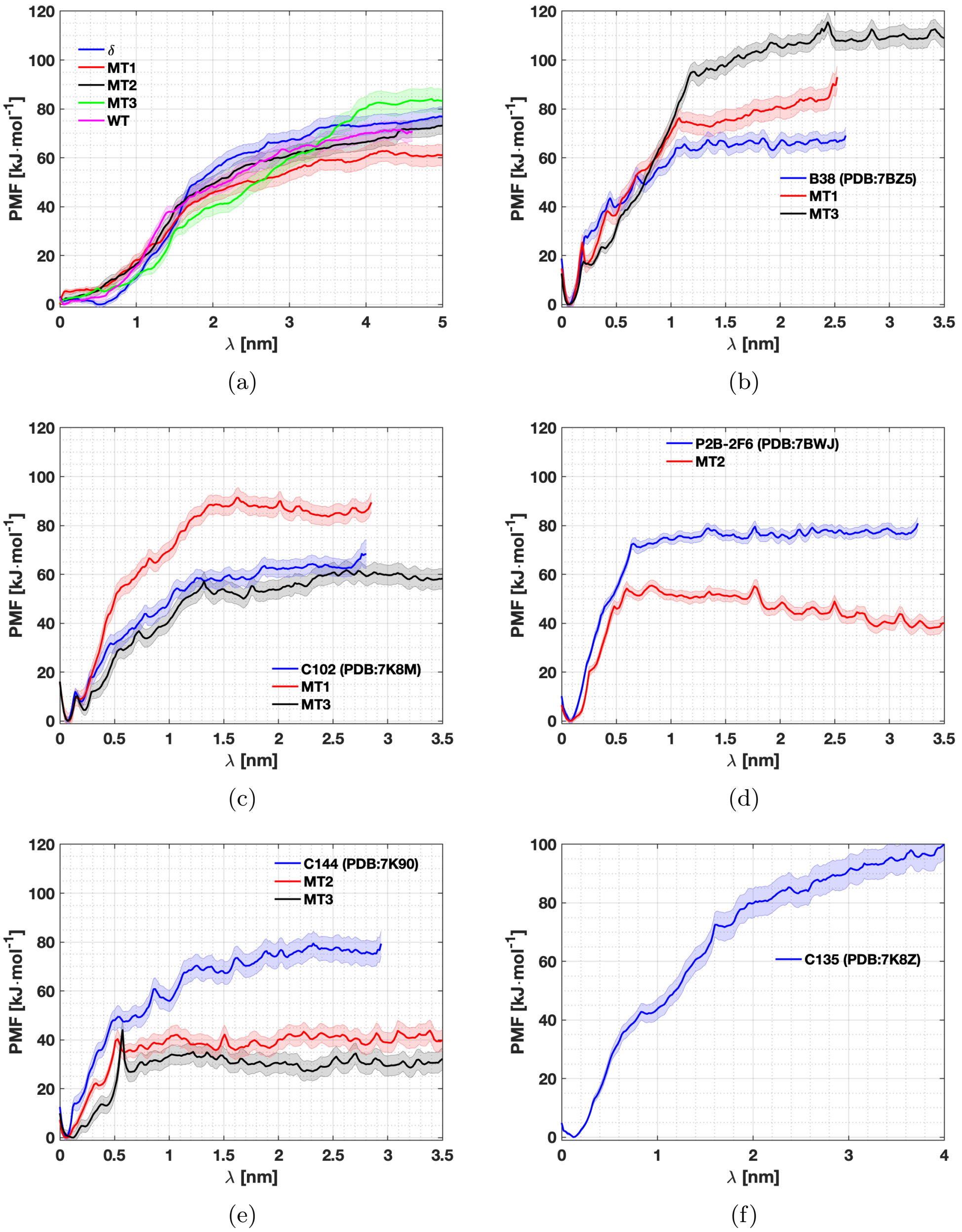

Figure S 1: PMF between the RBD and the ACE2 receptor and several hNAbs.

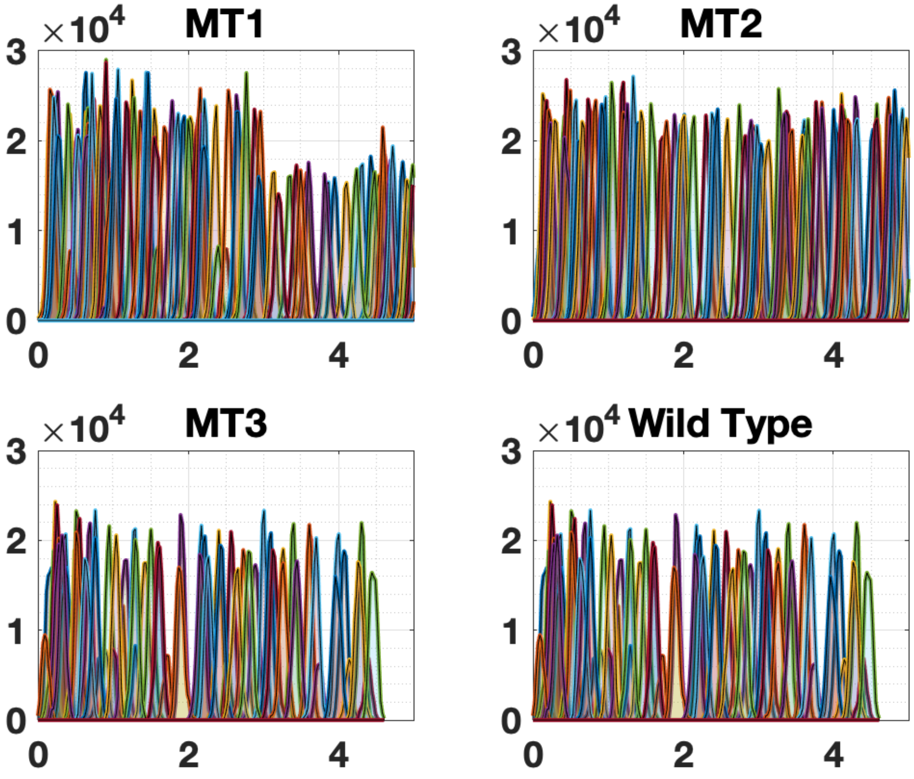

Figure S 2: Histogram showing the PMF sampling of the reaction coordinate for the RBD. The wild type (WT), MT1 (N501Y), MT2 (E484K, and N501Y), and MT3 (K417N, E484K, and N501Y) are shown.

2 Interactions between RBD and ACE2 receptor

Below we present the amino acid residues that are interacting between the RBD MT1 and the ACE2 receptor.

Table S 1: The hydrogen bonds and salt-bridges between SARS-CoV-2 RBD containing one mutation (N501Y) and labeled MT1 and the human ACE2 receptor.

$\lambda = 0 - 0.30$ nm	ACE2	Length(Å)	SARS-CoV-2 RBD
Hydrogen bond	D30	1.8	K417
	D38	1.6	Y449
	S19	1.8	A475
	S19	1.8	S477
	Y83	3.4	N487
	K31	1.9	Q493
	E35	2.3	Q493
	K353	1.9	G496
	K353	1.7	Q498
	K353	1.9	**Y501**
	N330	2.0	**Y501**
	D355	3.2	G502
	G354	2.9	V503
	G354	1.8	G504
	E37	1.8	Y505
Salt bridge	D30	2.7, 3.9	K417
$\lambda = 0.27 - 0.60$ nm			
Hydrogen bond	E23	1.8, 2.7	K458
	Y83	3.1	N487
	Y83	1.8	Y489
	K31	2.0	Q493
	K353	2.0	Y495
	K353	2.4	G496
	Q42	2.6	Q498
	R357	2.4, 3.2	T500
	K353	1.8	**Y501**
Salt bridge	E23	2.7	K458

Table S 2: The hydrogen bonds and salt-bridges between SARS-CoV-2 RBD containing one mutation (N501Y) and labeled MT1 and the human ACE2 receptor.

$\lambda = 0.62 - 0.85$ nm	ACE2	Length(Å)	SARS-CoV-2 RBD
Hydrogen bond	D30	1.7	K417
	H34	3.2	Y453
	Y83	1.7	N487
	Y83	2.0	Y489
	H34	3.2	Q493
	D38	2.8	Q493
	K353	1.8	Y495
	K353	3.4	G496
	Y41	2.2	Q498
	R357	1.9	T500
	K353	1.7	**Y501**
	D355	2.0	G502
Salt bridge	D30	2.6, 3.5	K417
$\lambda = 1.14 - 1.42$ nm			
Hydrogen bond	D30	2.3	K417
	Y83	1.8	N487
Salt bridge	D30	2.7	K417
$\lambda = 1.65 - 1.93$ nm			
Hydrogen bond	Q24	2.3	N487
	Y83	1.6, 2.8	N487
$\lambda = 2.83 - 3.1$ nm			
Hydrogen bond	S19	2.2	Y473
	Q24	2.8	Y475
	Y83	3.4	N487

Below we present the amino acid residues that are interacting between the RBD containing three mutations (K417N/E484K/N501Y) mutation and the human ACE2 receptor.

Table S 3: The hydrogen bonds and salt-bridges between SARS-CoV-2 RBD containing three mutations (K417N/E484K/N501Y) and labeled MT3 and the human ACE2 receptor.

$\lambda = 0 - 0.30$ nm	ACE2	Length(Å)	SARS-CoV-2 RBD
Hydrogen bond	R357	1.8, 3.2	T500
	K353	1.7	**Y501**
	D355	2.1	G502
$\lambda = 0.75 - 1.05$ nm			
Hydrogen bond	H34	2.6	Y453
	E35	1.8	**K484**
	Y83	2.1	N487
	N330	3.3	T500
	D355	3.3	T500
	K353	2.6	**Y501**
	K353	3.1	G502
	T324	2.7	V503
Salt bridge	E35	1.3	**K484**
$\lambda = 2.25 - 2.60$ nm			
Hydrogen bond	Y83	1.9	N487
$\lambda = 3.51 - 3.82$ nm			
Hydrogen bond	E35	1.7, 2.5	**K484**
Salt bridge	E35	2.1	**K484**

Table S 4: The hydrogen bonds and salt-bridges between SARS-CoV-2 RBD containing two mutations (L452R/T478K) and labeled δ and the human ACE2 receptor.

$\lambda = 0 - 0.30$ nm	ACE2	Length(Å)	SARS-CoV-2 RBD
Hydrogen bond	D30	1.6	K417
	S19	2.0	A475
	Q24	2.2	N487
	Y83	1.8	N487
	E35	1.8	Q493
	K353	2.7	G496
	K353	2.3	Q498
	Y41	1.8	T500
	K353	3.0	N501
	K353	2.0	G502
	E37	1.8,2.4	Y505
	R393	2.8	Y505
Salt bridge	D30	2.7,3.8	K417
$\lambda = 2.35 - 2.60$ nm			
Hydrogen bond	Y83	2.0	Y489
	K353	3.6	N501
$\lambda = 3.4 - 3.6$ nm			
Hydrogen bond	Y83	2.5	N487

3 Class I antibodies

Below we present the amino acid residues that are interacting between the RBD (in its WT and mutated versions) and with Class II antibodies B38 and C102.

Table S 5: The hydrogen and salt-bridges between SARS-CoV-2 RBD and B38 (PDB:7BZ5) antibody.

$\lambda = 0.02 - 0.13$ nm	Heavy Chain	Length(Å)	SARS-CoV-2 RBD	Length(Å)	Light Chain
Hydrogen bond			R403	1.7	N92
			E406	2.9	Y94
	Y52	2.7	K417	2.5	N92
	S56	1.6	D420		
	S53	3.3	Y421		
	G54	2.0	Y421		
			Y453	2.4	N92
	Y33	1.7	L455		
	S53	2.0	R457		
	S53	2.8	K458		
	S31	2.0	Y473		
	I28	2.3	A475		
	N32	2.2	A475		
	G26	1.7, 2.8	N487		
	R97	2.2, 2.5	N487		
	Y100	3.0, 3.2	F490		
			Q498	2.3	S30
			Q498	2.1	S67
			T500	3.2	G28
			N501	2.7	S30
			G502	1.9	G28
			V503	3.0	Q27
			Y505	2.6	Q90
			Y505	2.2	L91

Table S 6: The hydrogen and salt-bridges between SARS-CoV-2 RBD and B38 (PDB:7BZ5) antibody.

$\lambda = 0.06 - 0.19$ nm	Heavy Chain	Length(Å)	SARS-CoV-2 RBD	Length(Å)	Light Chain
Hydrogen bond			R403	1.8, 2.9	N92
			Q409	3.3	Y94
	Y58	2.7	T415		
			K417	1.8	N92
	G54	3.1	Y421		
	Y33	1.8	L455		
	N32	2.3	A475		
	G26	2.1	N487		
	R97	2.2, 2.5	N487		
	Y100	2.9	F490		
	Y100	2.9	Q493		
			Y495	1.9	Y32
			G496	2.0	S30
			Q498	2.7	S67
			T500	3.1	G28
			G502	1.9	G28
			Y505	2.0	Q90
			Y505	2.5	L91
			Y505	2.6	N92

Table S 7: The hydrogen and salt-bridges between SARS-CoV-2 RBD and B38 (PDB:7BZ5) antibody.

$\lambda = 0.09 - 0.31$ nm	Heavy Chain	Length(Å)	SARS-CoV-2 RBD	Length(Å)	Light Chain
Hydrogen bond			R403	1.8,2.2	N92
			E406	2.8	N92
	Y52	3.4	K417		
	G54	3.4	Y421		
			Y453	3.1	N92
	Y33	1.7	L455		
	S31	3.1	K458		
	S31	2.0	Y473		
	I28	1.9	A475		
	N32	2.9	A475		
	G26	2.9	N487		
	R97	2.2, 2.9	N487		
	R97	2.8	Y489		
	Y100	3.3	F490		
	Y100	3.0	Q493		
			S494	2.9	Y32
			Y495	1.9	Y32
			G496	1.9	S30
			Q498	2.4	S30
			Q498	2.6	S67
			T500	3.4	G28
			N501	2.9	S30
			G502	2.0	G28
			Y505	1.9	Q90
			Y505	2.3	L91

Table S 8: The hydrogen and salt-bridges between SARS-CoV-2 RBD and B38 (PDB:7BZ5) antibody.

$\lambda = 0.67 - 0.89$ nm	Heavy Chain	Length(Å)	SARS-CoV-2 RBD	Length(Å)	Light Chain
	N32	2.5	A475		
	I28	1.9	A475		
	G26	2.3	N487		
	R97	1.8, 2.5	N487		
$\lambda = 0.99 - 1.27$ nm					
Hydrogen bond	S31	1.9	N487		

Table S 9: The hydrogen bonds and salt-bridges between SARS-CoV-2 RBD containing one mutation (N501Y) labeled as MT1 in the manuscript and the hNAbs B38 (PDB:7BZ5).

$\lambda = 0.02 - 0.14$ nm	Heavy Chain	Length(Å)	SARS-CoV-2 RBD	Length(Å)	Light Chain
Hydrogen bond			R403	1.9, 2.6	N92
	Y58	2.6, 2.8	T415		
	S56	3.1	G416		
	Y52	3.3	K417	2.1	N92
	S56	1.6	D420		
	S53	3.2	Y421		
	G54	2.1	Y421		
	Y33	1.6	L455		
	S53	1.9	R457		
	S31	3.2	K458		
	S31	1.9	Y473		
	I28	2.2	A475		
	N32	1.9	A475		
	G26	1.9	N487		
	R97	1.7, 2.2	N487		
	R97	3.1	Y489		
	Y100	3.0, 3.2	F490		
			G502	1.8	G28
			Y505	2.5	Q90
			Y505	1.9	L91

Table S 10: The hydrogen bonds and salt-bridges between SARS-CoV-2 RBD containing one mutation (N501Y) labeled as MT1 in the manuscript and the hNAbs B38 (PDB:7BZ5).

$\lambda = 0.19 - 0.34$ nm	Heavy Chain	Length(Å)	SARS-CoV-2 RBD	Length(Å)	Light Chain
Hydrogen bond	S56	3.0	G416		
			K417	3.1	N92
	S56	3.4	D420		
	S53	2.8	Y421		
	G54	1.9	Y421		
	Y33	1.9	L455		
	S53	2.1	R457		
	S30	2.5	K458		
	G54	2.5	K460		
	S31	2.2	Y473		
	I28	2.2	A475		
	N32	2.6	A475		
	Y100	1.6	E484		
	G26	2.4	N487		
	Y100	2.9	F490		
			S494	3.3	Y32

Table S 11: The hydrogen bonds and salt-bridges between SARS-CoV-2 RBD containing one mutation (N501Y) labeled as MT1 in the manuscript and the hNAbs B38 (PDB:7BZ5).

$\lambda = 0.22 - 0.53$ nm		Heavy Chain	Length(Å)	SARS-CoV-2 RBD	Length(Å)	Light Chain
Hydrogen bond	S56	2.8	G416			
	Y52	3.0	K417	3.1	N94	
	S56	1.8	D420			
	G54	1.9	Y421			
			Y453	3.1	N92	
	Y33	1.7	L455			
	S53	1.6, 2.6	R457			
	S30	2.4	K458			
	S31	2.0	K458			
	S31	1.8	Y473			
	I28	2.2	A475			
	N32	2.7	A475			
	G26	2.6	N487			
	R97	1.8, 2.0	N487			
	Y100	2.2	F490			
			Q493	2.5	Y32	
			**Y501**	2.7	S30	

Table S 12: The hydrogen bonds and salt-bridges between SARS-CoV-2 RBD containing one mutation (N501Y) labeled as MT1 in the manuscript and the hNAb B38 (PDB:7BZ5).

$\lambda = 0.78 - 0.99$ nm	Heavy Chain	Length(Å)	SARS-CoV-2 RBD	Length(Å)	Light Chain
Hydrogen bond	I28	2.3	A475		
	N32	2.6	A475		
	G26	2.0	N487		
	R97	1.9, 2.8	N487		
$\lambda = 1.37 - 1.57$ nm					
Hydrogen bond	S53	2.1	A475		
	N73	3.1	S477		
	S31	2.9	T478		
	S31	2.3	N487		
$\lambda = 1.55 - 1.80$ nm					
Hydrogen bond	S31	2.8	N487		

Table S 13: The hydrogen bonds and salt-bridges between SARS-CoV-2 RBD containing three mutations (K417N/E484K/N501Y) labeled as MT3 in the manuscript and the hNAb B38 (PDB:7BZ5).

$\lambda = 0.02 - 0.13$ nm	Heavy Chain	Length(Å)	SARS-CoV-2 RBD	Length(Å)	Light Chain
Hydrogen bond			R403	2.0, 2.1	N92
	Y58	2.4	T415		
	S56	1.6	D420		
	S53	1.8, 2.8	Y421		
	G54	2.9	Y421		
			Y453	2.6	N92
	Y33	2.0	L455		
	S31	2.1	Y473		
	I28	2.6	A475		
	N32	2.0	A475		
	G26	2.6	N487		
	R97	1.9, 1.9	N487		
	R97	3.1	Y489		
	Y100	3.2	F490		
			G502	2.0	G28
			Y505	1.9	Q90
			Y505	2.1	L91

$\lambda = 1.37 - 1.57$ nm					
Hydrogen bond			R403	1.9, 2.3	N92
			Q409	3.1	Y94
	Y33	2.1	**N417**		
			Y453	2.7	N92
			L455	1.9	Y33
	S31	2.0	Y473		
	I28	2.4	A475		
	N32	2.1	A475		
	G26	2.2	N487		
	R97	1.9, 3.0	N487		
	Y100	2.6	F490		
	Y100	2.8	Q493		
			G496	2.3	S30
			Q498	2.7	S67
			T500	3.1	G28
			G502	2.0	G28
			V503	3.1	Q27
			Y505	2.1	Q90
			Y505	1.7	L91

Table S 14: The hydrogen and salt-bridges between SARS-CoV-2 RBD and C102 (PDB: 7K8M) antibody.

$\lambda = 0.02 - 0.13$ nm	Light Chain	Length(Å)	SARS-CoV-2 RBD	Length(Å)	Heavy Chain
Hydrogen bond	G92	2.8	R404		
			G416	3.1	S56
			K417	1.8	G97
			D420	1.7	S56
			Y421	2.2	G54
			L455	1.8	Y33
			R457	2.2	S53
			K458	3.3	S30
			N460	2.9	G54
			Y473	1.6	S31
			A475	2.0	I28
			A475	2.1	N32
	S30	2.8	N501		
	S29	3.1	G502		
	Y32	2.0	Y505		

Table S 15: The hydrogen and salt-bridges between SARS-CoV-2 RBD and C102 (PDB: 7K8M) antibody.

$\lambda = 0.10 - 0.27$ nm	Light Chain	Length(Å)	SARS-CoV-2 RBD	Length(Å)	Heavy Chain
Hydrogen bond			K417	3.6	Y52
			K417	1.7	G97
			D420	1.5	S56
			Y421	3.0	Y33
			Y421	1.8	G54
			Y453	2.4	Y99
			L455	1.9	Y33
			R457	1.8, 2.7	S53
			K458	3.0	S31
			K458	3.9	G54
			N460	3.0	S56
			Q474	3.6	S31
			A475	1.8	I28
			A475	2.5	N32
			Y489	2.7	R94
			Q493	1.6	Y99

Table S 16: The hydrogen and salt-bridges between SARS-CoV-2 RBD and C102 (PDB: 7K8M) antibody.

$\lambda = 0.36 - 0.60$ nm	Light Chain	Length(Å)	SARS-CoV-2 RBD	Length(Å)	Heavy Chain
Hydrogen bond			R457	2.0	S53
			Y473	2.3	S31
			A475	2.8	I28
			A475	1.9	N32
			S477	1.9	G26
			Y489	2.2	R94
$\lambda = 0.78 - 0.97$ nm					
Hydrogen bond			A475	2.1	I28
			A475	2.2	N32
			S477	2.2	G26
			N487	3.2, 3.4	R94
			Y489	3.1	R94
$\lambda = 1.26 - 1.52$ nm					
Hydrogen bond			N487	3.0, 3.1	S31
$\lambda = 1.64 - 1.87$ nm					
Hydrogen bond			S477	1.8, 2.8	G54
			N487	1.8, 3.3	S53

Table S 17: The hydrogen bonds and salt-bridges between SARS-CoV-2 RBD containing one mutation (N501Y) and labeled MT1 and the hNAb C102 (PDB: 7K8M).

$\lambda = 0 - 0.13$ nm	Light Chain	Length(Å)	SARS-CoV-2 RBD	Length(Å)	Heavy Chain
Hydrogen bond	G92	3.3	R403		
			K417	2.2	G97
			D420	1.7	S56
			Y421	2.1	G54
			Y421	2.9	G55
			Y453	2.1	Y99
			L455	2.0	Y33
			R457	1.6, 2.8	S53
			Y473	1.7	S31
			A475	2.1	I28
			A475	2.1	N32
	S29	2.5	G502		
	S30	3.4	Y505		

Table S 18: The hydrogen bonds and salt-bridges between SARS-CoV-2 RBD containing one mutation (N501Y) and labeled MT1 and the hNAb C102 (PDB: 7K8M).

$\lambda = 0.05 - 0.23$ nm	Light Chain	Length(Å)	SARS-CoV-2 RBD	Length(Å)	Heavy Chain
Hydrogen bond	G92	2.4	R403		
			K417	1.7	G97
			D420	1.7	S56
			Y421	2.1	G54
			L455	2.0	Y33
			R457	2.0, 2.9	S53
			Y473	1.9	S31
			A475	2.5	I28
			A475	2.3	N32
			Y489	2.7	R94
	S29	2.8	G502		
	S30	2.2	Y505		

Table S 19: The hydrogen bonds and salt-bridges between SARS-CoV-2 RBD containing one mutation (N501Y) and labeled MT1 and the hNAb C102 (PDB: 7K8M).

$\lambda = 0.15 - 0.33$ nm	Light Chain	Length(Å)	SARS-CoV-2 RBD	Length(Å)	Heavy Chain
Hydrogen bond			K417	1.8	G97
			D420	2.6	S56
			Y421	2.2	G54
			Y421	3.3	S56
			L455	1.9	Y33
			R457	2.0, 3.1	S53
			N460	2.9	G54
			Y473	2.2	S31
			Q474	3.2	S31
			A475	2.3	I28
			A475	1.9	N32
	Y32	3.2	Q493		
			Q493	2.8	Y99
	G92	1.8	Y505		

Table S 20: The hydrogen bonds and salt-bridges between SARS-CoV-2 RBD containing one mutation (N501Y) and labeled MT1 and the hNAb C102 (PDB: 7K8M).

$\lambda = 0.27 - 0.53$ nm	Light Chain	Length(Å)	SARS-CoV-2 RBD	Length(Å)	Heavy Chain
Hydrogen bond			K417	1.6	G97
			D420	1.9	S56
			Y421	2.5	Y33
			Y421	2.5	S53
			L455	1.7	Y33
			R457	1.9, 3.0	S53
			N460	2.5	G56
			Y473	1.7	S31
			A475	1.9	I28
			A475	1.9	N32
			A477	1.9,3.5	G26
			Y489	1.8, 2.9	R94
	Y32	2.3	Q493		
			Q493	1.7, 3.1	Y99
$\lambda = 0.83 - 1.1$ nm					
Hydrogen bond			A475	2.2	I28
			A475	2.5	N32
			A477	1.9,2.8	G26
$\lambda = 0.94 - 1.18$ nm					
Hydrogen bond			Y473	3.2	S31
			A475	2.2	I28
			A475	2.1	N32
			A477	1.8,2.5	G26

Table S 21: The hydrogen bonds and salt-bridges between SARS-CoV-2 RBD containing three mutations (K417N/E484K/N501Y)and labeled MT3 and the hNAb C102 (PDB: 7K8M).

$\lambda = 0.02 - 0.13$ nm	Light Chain	Length(Å)	SARS-CoV-2 RBD	Length(Å)	Heavy Chain
Hydrogen bond			T415	3.0	S56
			**N417**	2.0, 3.6	Y33
			**N417**	2.8	G97
			D420	1.8	S56
			Y421	2.5	G54
			Y421	2.8	G55
			Y421	3.1	S56
			R457	1.6, 2.9	S53
			R458	2.6	S31
			N460	1.9	S56
			Y473	1.8	S31
			Q474	3.3	S31
			A475	2.3	I28
			A475	1.8	N32
			Y489	2.9	R94
	S29	3.1	G502		
	Y32	3.4	Y505		
$\lambda = 0.7 - 0.9$ nm					
Hydrogen bond			**N417**	2.7	Y33
			D420	1.8	S56
			Y421	1.9	G54
			Y453	2.7	Y99
			L455	1.6	Y33
			R457	2.2	S53
			Y473	1.8	S31
			A475	2.1	I28
			A475	1.9	N32

4 Class II antibodies

Below we present the amino acid residues that are interacting between the RBD (in its WT and mutated versions) and with Class II antibodies P2B-2F6 and C144.

Table S 22: The hydrogen bonds and salt-bridges between SARS-CoV-2 RBD and P2B-2F6 (PDB: 7BWJ) antibody

$\lambda = 0 - 0.14$ nm	Heavy chain	Length(Å)	SARS-CoV-2 RBD	Length(Å)	Light chain
Hydrogen bond	Y27	1.9	G447		
	S31	1.9	Y449		
	S30	1.8	N450		
	H54	1.9	N450		
			G482	3.3	Y34
	R112	1.8, 3.2	E484	2.4	N33
			E484	2.6	Y34
Salt bridge	R112	2.0, 3.7	E484		
$\lambda = 0.62 - 0.87$ nm					
Hydrogen bond	R112	1.7, 2.4	E484		
			G482	1.9	Y34
Salt bridge	R112	0.8, 1.7	E484		
$\lambda = 0.98 - 0.76$ nm					
Hydrogen bond	R112	2.6, 3.1	E484		
			G482	3.4	Y34
Salt bridge	R112	3.1	E484		
$\lambda = 1.15 - 1.4$ nm					
Hydrogen bond			E484	2.3	Y34

Table S 23: The hydrogen bonds and salt-bridges between SARS-CoV-2 RBD and C144 (PDB: 7K90) antibody.

$\lambda = 0 - 0.12$ nm	Light Chain	Length(Å)	SARS-CoV-2 RBD	Length(Å)	Heavy Chain
Hydrogen bond			K417	3.0	Y100
			E484	1.7, 2.2	S53
			E484	1.9	G54
			E484	2.1, 2.3	S56
			E484	3.1	R100
			Y489	1.7	D100
			F490	1.8, 3.2	R100
			L492	2.6	R100
			Q493	1.8, 2.6	R100
			S494	3.5	S30
			S494	1.9, 2.2, 2.8, 3.2	N31
$\lambda = 0.32 - 0.48$ nm					
Hydrogen bond			E484	1.8	S53
			E484	3.5	S56
			E484	1.9, 2.7	R100
			Y489	1.9, 2.5	D100
Salt bridge			E484	3.5	R100
$\lambda = 0.53 - 0.75$ nm					
			F486	3.2	S56
$\lambda = 0.93 - 1.14$ nm					
Hydrogen bond			F486	1.8	G54

Table S 24: The hydrogen bonds and salt-bridges between SARS-CoV-2 RBD containing three mutations (K417N/E484K/N501Y) and labeled MT3 and the hNAbs C144 (PDB: 7K90).

$\lambda = 0 - 0.12$ nm	Light Chain	Length(Å)	SARS-CoV-2 RBD	Length(Å)	Heavy Chain
Hydrogen bond			Y449	2.8	G26
			**K484**	2.6	S55
	Y91	3.0	F486		
			N487	3.0	D100
			F490	2.0, 2.8	R100
			L492	2.6	R100
			Q493	1.9, 2.4	R100
			S494	2.1, 2.1	N31
			Q498	2.9	G26
$\lambda = 0.7 - 0.9$ nm					
Hydrogen bond			**N417**	2.3	Y100
			Y489	2.4	R100
			Q493	2.6	R100

5 Class III antobodies

Below we present the amino acid residues that are interacting between the RBD and with Class III antibody C135.

Table S 25: The hydrogen bonds and salt-bridges between SARS-CoV-2 RBD and C135 (PDB: 7K8Z) antibody.

$\lambda = 0.07 - 0.29$ nm	Heavy Chain	Length(Å)	SARS-CoV-2 RBD	Length(Å)	Light Chain
Hydrogen bond	Y98	3.4	T345		
			T345	2.4	Y94
	R55	2.1	N437		
	D53	1.9	N440		
	N56	2.6	N440		
	S96	1.9	V445		
			Y451	3.2	W-32
$\lambda = 0.26 - 0.55$ nm					
Hydrogen bond			T345	1.9	N92
	R55	1.9, 2.8	S438		
	D53	1.8	N440		
	N56	2.2	N440		
	S96	2.9	V445		
$\lambda = 0.34 - 0.61$ nm					
Hydrogen bond			T345	1.9	N92
	R55	2.2, 2.3	S438		
	D53	1.7, 2.6	N440		
	N56	2.2	N440		
	S96	1.9	V445		
$\lambda = 0.36 - 0.68$ nm					
Hydrogen bond			T345	1.9	N92
$\lambda = 0.59 - 0.86$ nm					
Hydrogen bond			T345	2.0	N92
	S96	1.9	V445		
$\lambda = 0.91 - 1.23$ nm					
Hydrogen bond			T345	1.7, 2.2, 3.1	E50
	S96	2.3,3.2	V445		
$\lambda = 1.05 - 1.35$ nm					
Hydrogen bond	G97	1.8	T345		
			R346	1.7, 2.2	E50
